# Mitochondrial polarization and redox homeostasis couple glycolysis-to-OXPHOS metabolic rewiring to lifespan extension in *C. elegans*

**DOI:** 10.17912/micropub.biology.001858

**Published:** 2025-11-19

**Authors:** Anwaar Ali, Shanmuganathan Balakrishnan, Jiayu Xiang, Haesoo Bae, Minou Tsujishita, Amina Abulimiti, Bence Nemeth, Michalis Barkoulas, Kambiz N Alavian

**Affiliations:** 1 Department of Brain Sciences, Faculty of Medicine, Imperial College London, London, England, United Kingdom; 2 Department of Life Sciences, Faculty of Natural Sciences, Imperial College London, London, England, United Kingdom

## Abstract

Mitochondria are essential for maintaining cellular homeostasis throughout life. Here, we investigated the differential effects of glucose and galactose, as well as glycolytic inhibition, on *C. elegans* lifespan in relation to mitochondrial membrane potential and reactive oxygen species (ROS) levels. Our results show that long-term treatment with glucose reduces both lifespan and mitochondrial membrane potential, whereas galactose increases them. The increase in mitochondrial membrane potential and lifespan is inversely correlated with mitochondrial ROS levels, suggesting a role for mitohormesis in lifespan extension.

**Figure 1. Effects of glucose, galactose, and 2-DG on C. elegans lifespan, mitochondrial membrane potential (Δψm), and mitochondrial ROS:**
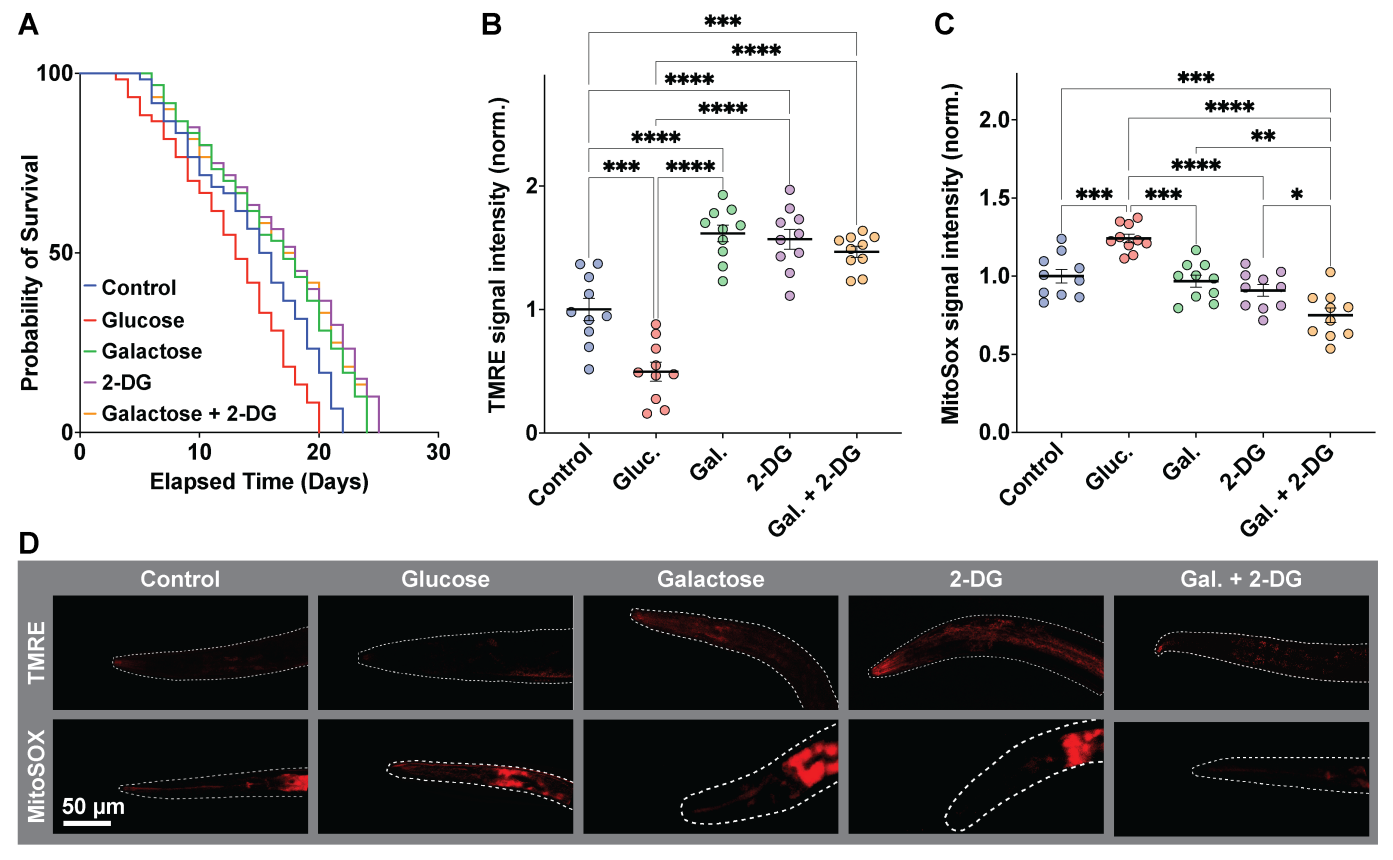
**A.** Kaplan-Meier survival curves for control (unsupplemented media), glucose, galactose, 2-DG, and galactose + 2-DG, n = 60 per group; overall differences by log-rank test p < 0.0001 and Gehan-Breslow-Wilcoxon test p < 0.0001. Median lifespan (days): control 15.5, glucose 13.0, galactose 17.0, 2-DG 18.0, galactose + 2-DG 17.5. Pairwise versus control by log-rank: glucose p = 0.0041, galactose p = 0.0134, 2-DG p = 0.0013, galactose + 2-DG p = 0.0052. **B.** TMRE fluorescence in day 5 adults, normalized to control, n = 10 per group; one-way ANOVA with Tukey's test: control vs glucose p = 0.0002; glucose vs galactose, 2-DG, and galactose + 2-DG all p < 0.0001; control vs galactose p < 0.0001; control vs 2-DG p < 0.0001; control vs galactose + 2-DG p = 0.0005; galactose vs 2-DG p = 0.9913; galactose vs galactose + 2-DG p = 0.6091; 2-DG vs galactose + 2-DG p = 0.8601. **C.** MitoSOX Red fluorescence in day 5 adults, normalized to control, n = 10 per group; one-way ANOVA with Tukey's test: versus glucose-control p = 0.0007, galactose p = 0.0001, 2-DG p < 0.0001, galactose + 2-DG p < 0.0001; galactose + 2-DG lower than control p = 0.0004, galactose p = 0.0024, and 2-DG p = 0.0463; control vs galactose p = 0.9767, control vs 2-DG p = 0.4693, galactose vs 2-DG p = 0.8188. D. Representative TMRE top and MitoSOX bottom micrographs from day 5 adults; quantification in panels B and C.

## Description

Perturbations in cellular homeostasis and a progressive decline in physiological integrity are hallmarks of aging. Mitochondrial dysfunction is also a fundamental driver of aging and a wide range of aging-related processes (Srivastava, 2017). Mitochondrial membrane potential (Δψm) is the voltage across the inner mitochondrial membrane established primarily by electron transport chain proton pumping which powers ATP synthesis and metabolite transport, maintaining optimal metabolic function (Nicholls, 2004). Evidence points towards age-related decline in Δψm, observed in multiple aging models, including *C. elegans* , reflecting impaired ETC function (Berry et al., 2023; Mansell et al., 2021). Mild mitochondrial depolarization has been shown to paradoxically extend lifespan, likely by promoting systemic homeostasis through upregulation of stress response mechanisms (Hong et al., 2024).

The effects of glucose on metabolism are dichotomous. On the one hand, it fuels oxidative phosphorylation (OXPHOS) and ATP production. On the other, chronic hyperglycaemia has been shown to lower Δψm and disturb mitochondrial respiration (Chia et al., 2018). Studies in *C. elegans* have shown that glucose-enriched diets significantly shorten lifespan through increase of insulin/IGF-1 signalling pathway. The worms also showed an increase in lipoperoxidation and oxidative stress, caused by the elevated expression of *sod-3* (Lee et al., 2009; Schulz et al., 2007) (Alcántar-Fernández et al., 2018).

2-deoxyglucose (commonly 2-DG), a glucose analogue and competitive inhibitor of glycolysis, acts as a dietary-restriction mimetic, inducing pathways similar to those activated under caloric restriction (Ingram et al., 2006). In *C. elegans* , chemical attenuation of glycolysis with 2-DG shifts metabolic dependence towards OXPHOS, increasing mitochondrial ATP production and oxygen consumption to compensate for reduced glycolytic ATP, extending lifespan (Schulz et al., 2007). While the mechanism linking inhibition of glycolysis to longevity remains unclear, reduced glucose availability - with consequent increases in ETC activity and OXPHOS - is known to elevate reactive oxygen species (ROS) levels. This metabolic shift has been hypothesized to trigger an adaptive, hormesis-dependent response to moderately elevated ROS, culminating in globally upregulated endogenous stress-response mechanisms and extended lifespan (Ristow & Schmeisser, 2014) (Bárcena et al., 2018).

Similar to 2-DG, galactose supplementation modulates intracellular glucose levels and metabolic flux, promoting a metabolic shift towards OXPHOS (Aguer et al., 2011). The Leloir pathway is the most common route for galactose metabolism, where galactose is converted to glucose-6-phosphate through a series of enzymatic reactions, utilizing galactose mutarotase, galactokinase (GALK), galactose-1-phosphate uridylyltransferase (GALT), and UDP-galactose 4-epimerase to facilitate an entry point for glycolytic catabolism (Holden et al., 2003). It is not, however, clear whether this shift toward OXPHOS results in extension of lifespan, as galactose induces oxidative stress, mimicking the aging process in *D. melanogaster* and rodent models (Cui et al., 2006). Emerging data in *C. elegans* have shown that the core enzymatic components of the Leloir pathway are conserved, and have confirmed the lethal effect of complete loss-of-function of the GALE homolog, *gale-1* . However, galactose metabolic mechanism in *C. elegans* , and the extent to which galactose-induced metabolic stress falls within a hormetic threshold are yet to be elucidated (Aguer et al., 2011; Watts & Ristow, 2017).

To assess the effect of carbohydrate source and glycolytic inhibition on mitochondrial membrane potential and lifespan, *C. elegans* synchronized embryos obtained by alkaline hypochlorite bleaching were reared continuously from hatching on media supplemented with 10 mM glucose, 2.5 mM galactose, 5 mM 2-DG, along with unsupplemented control media. Survival was different across these treatments by log-rank (X² = 40.12, df = 4, p < 0.0001) and Gehan-Breslow-Wilcoxon tests (X² = 24.30, df = 4, p < 0.0001). A significant trend toward progressively longer lifespan was observed in conditions that shift metabolism away from glycolysis (log-rank trend X² = 21.97, df = 1, p < 0.0001), with median lifespan on glucose at 13.0 days, in control media at 15.5 days, and 17.0, 18.0, and 17.5 days on galactose, 2-DG, and galactose + 2-DG, respectively ( Figure 1A )(n = 60 per condition, no censoring).

To assess whether there is a correlation between lifespan and mitochondrial membrane potential, we quantified Δψm with tetramethylrhodamine ethyl ester (TMRE) 8 days after bleaching (adult day 5). In comparison to the control group mean value, glucose treated worms had the lowest TMRE intensity (M = 0.496 ± 0.074), while galactose, 2-DG and galactose + 2-DG treatments all increased membrane potential in comparison to the control or glucose-treated nematodes (TMRE M = 1.616 ± 0.074, 1.570 ± 0.074, and 1.467 ± 0.074, respectively - all Tukey - adjusted p < 0.0005). No difference was observed among the three latter treatment groups (all p ≥ 0.60), ( Figure 1B, D). Median lifespan correlated closely with normalized Δψm at the group level (Pearson r = 0.97), indicating higher Δψm with longer survival relative to glucose.

Previous studies in *C. elegans* show that glucose supplementation increases oxidative stress and shorten lifespan, whereas dietary restriction and attenuating glycolysis with 2-DG can extend lifespan through mitohormetic ROS signalling. To test whether the lifespan and Δψm differences we observed align with this scenario, we quantified mitochondrial ROS in day 5 adults using MitoSOX Red. Relative (normalized) to control worms maintained in unsupplemented media (control), glucose-treated animals showed the highest mitochondrial ROS (M = 1.241 ± 0.055), whereas MitoSOX intensity was decreased in galactose, 2-DG, and galactose + 2-DG-treated groups (0.968 ± 0.055, 0.908 ± 0.055, and 0.749 ± 0.055, respectively; n = 10 per group) ( Figure 1C, D). These results collectively suggest that diverting carbon flux toward OXPHOS extends lifespan by increasing the coupling of ETC (reduced electron leak), potentially triggering a mitohormetic upregulation of antioxidant and mitochondrial quality control pathways.

## Methods

***C. elegans* maintenance and synchronisation**

All experiments were conducted using wild-type *C. elegans* N2 strain, obtained from *Caenorhabditis* Genetics Center (CGC), University of Minnesota. Worms in the control group were maintained at 20°C on unsupplemented nematode growth medium (NGM) plates seeded with OP50 *E. coli* . Age-synchronous populations were obtained through standard hypochlorite bleaching of gravid adults.

**Lifespan Assay**

Synchronized L1 larvae were cultured on NGM plates until day one of adulthood. At the onset of adulthood, 20 worms per condition were transferred to liquid medium supplemented with 100 µM 5-fluoro-2'-deoxyuridine (FUdR), streptomycin, and *E. coli* OP50 . Worms were treated daily with 10 mM glucose, 2.5 mM galactose, 5 mM 2-deoxyglucose (2-DG), or a combination of galactose and 2-DG. Each experiment was performed in triplicate. Worm viability was assessed daily under a dissecting microscope; worms that did not respond to gentle prodding with a worm pick were scored as dead, and those with progeny retention or extruded gonads were omitted from subsequent analysis. All the chemicals were from Millipore (Sigma-Aldrich).

**Measurement of mitochondrial membrane potential and superoxide production**

Day 5 adult worms (8 days post-bleaching), treated daily as described above, were collected in M9 buffer and washed three times by centrifugation before incubation with either 20 nM tetramethylrhodamine ethyl ester (TMRE; Thermo Fisher Scientific) or 1 μM MitoSOX Red (Thermo Fisher Scientific) for 30 minutes at room temperature. Following staining, worms were immobilized with 100 μM tetramisole hydrochloride. Fluorescence was quantified from images acquired under fixed exposure parameters on a widefield imaging system. Illustrative high-resolution confocal micrographs were captured using a Leica SP8 microscope. Fiji (ImageJ - version 2.16.0/1.54p) was used for quantification and background subtraction of whole-worm fluorescence intensity. Data were analyzed using GraphPad Prism 10.
